# Supplementary material for: Prospective study of Candida auris nucleic acids in wastewater solids in 190 wastewater treatment plants in the United States suggests widespread occurrence
Source: mBio. 2024 Jul 23;15(8):e00908-24. doi: 10.1128/mbio.00908-24 (PMC11323724; doi:10.1128/mbio.00908-24)
Supplement: Supplemental Material — Supplemental figures, tables, and text. [file mbio.00908-24-s0001.pdf]

Supporting information for

Prospective study of *Candida auris* nucleic-acids in wastewater solids in 190 wastewater treatment plants in the United States suggests widespread occurrence

Alessandro Zulli[1], Elana M. G. Chan[1], Bridgette Shelden[2], Dorothea Duong[2], Xiang-Ru S. Xu[2], Bradley J. White[2], Marlene K. Wolfe[3], Alexandria B. Boehm[1*]

1. Department of Civil and Environmental Engineering, Stanford University, 473 Via Ortega, Stanford, California, 94305
2. Verily Life Sciences LLC, South San Francisco, CA, USA
3. Gangarosa Department of Environmental Health, Rollins School of Public Health, Emory University, 1518 Clifton Rd, Atlanta, GA, USA, 30322

*corresponding author: aboehm@stanford.edu

Additional details related to EMMI guidelines. One-hundred forty (140) wastewater samples from the study were selected at random for this analysis; this represents ~10% of the samples processed in the study. The randomly selected samples were from the samples for which 10 replicate wells were run for C. auris as these raw data were relatively more easily accessible. The average (standard deviation) number of partitions (droplets) for the across 10 replicate wells was 173,777 (29,518) for the reaction for *C. auris*. The volume of the partitions, as reported by the machine vendor is 0.00085 µL. The average (standard deviation) of copies per partition for the C. auris target is 1.37×10^{-6} (3.14×10^{-6}). An example fluorescent plot from the QX600 (6 color reader) is included in the Stanford Digital Repository with the deposited wastewater data (https://purl.stanford.edu/hv291tt0888).

Table S1. Plant characteristics. Plants marked with an asterisk (*) are plants where 10 replicate wells were used for digital droplet PCR (SCAN plants in caption of Table S2), the remaining plants had 6 wells used to measure C. auris, and 2 for PMMoV and BCoV. The site name, location (town, and state), state where plant is located, and whether the sample originally supplied to us was a 24 hour composite sample of liquid influent (“liquid”) or a sample from the primary clarifier (“solids”). Regardless of how the samples were provided, the solids were processed for analysis as described in the methods section. The population served is also provided.

Site Name	Location	State	Sample Type	Population served
Valley Creek Water Reclamation Facility	Bessemer, AL	Alabama	Liquids	225,000
Cahaba River Water Reclamation Facility	Cahaba River, Birmingham, AL	Alabama	Liquids	95,000
Five Mile Creek Water Reclamation Facility	Fultondale, AL	Alabama	Liquids	77,000
Turkey Creek Water Reclamation Facility	Pinson, AL	Alabama	Liquids	30,000
Village Creek Water Reclamation Facility	Village Creek, Birmingham, AL	Alabama	Liquids	200,000
John M. Asplund Water Pollution Control Facility	Anchorage, AK	Alaska	Liquids	220,000
City of Harrison Wastewater Treatment Plant	Harrison, AR	Arkansas	Liquids	15,000
Coastal Treatment Plant	Coastal, Laguna Niguel, CA	California	Liquids	48,000
Central Contra Costa Sanitary District	Contra Costa County, CA	California	Solids	484,800
City of Davis Wastewater Treatment Plant	Davis, CA	California	Solids	68,000
Esparto Wastewater Treatment Facility	Esparto, CA	California	Liquids	4,006
Fairfield-Suisun Sewer District	Fairfield, CA	California	Solids	155,000
[Fremont Basin] - Raymond A. Boege Alvarado WWTP	Fremont, CA	California	Liquids	229,476

South County Regional Wastewater Authority *	Gilroy, CA	California	Solids	110,338
Sewer Authority Mid-Coastside	Half Moon Bay, CA	California	Liquids	28,000
City of Hollister Domestic Water Recycling Facility	Hollister, CA	California	Liquids	42,000
Valley Sanitary District	Indio, CA	California	Solids	91,765
JB Latham Treatment Plant	JB Latham, Laguna Niguel, CA	California	Liquids	120,000
Lancaster Water Reclamation Plant	Lancaster, CA	California	Liquids	200,000
Las Gallinas Valley Sanitary District	Las Gallinas, San Rafael, CA	California	Liquids	30,000
Lompoc Regional Wastewater Reclamation Plant	Lompoc, CA	California	Liquids	69,290
Joint Water Pollution Control Plant	Los Angeles County, CA	California	Liquids	3,500,000
Hyperion Water Reclamation Plant (HWRP)	Los Angeles, CA	California	Liquids	4,000,000
Los Banos Wastewater Treatment Plant	Los Banos, CA	California	Liquids	42,000
City of Madera, Wastewater Treatment Plant	Madera, CA	California	Solids	67,944
Mammoth Community Water District	Mammoth, CA	California	Liquids	35,000
Monterey One Water - Regional Treatment Plant	Marina, CA	California	Liquids	262,000
Merced Wastewater Treatment Plant	Merced, CA	California	Solids	91,000
Sewerage Agency of Southern Marin Wastewater Treatment Plant	Mill Valley, CA	California	Solids	30,000
Modesto's Sutter Primary Treatment Facility	Modesto, CA	California	Solids	230,000
Soscol Water Recycling Facility	Napa, CA	California	Solids	83,300
[Newark Basin] - Raymond A. Boege Alvarado WWTP	Newark, CA	California	Liquids	47,229
Novato Sanitary District	Novato, CA	California	Liquids	53,000

East Bay Municipal Utility District	Oakland, CA	California	Solids	740,000
Oceanside Water Pollution Control Plant *	Oceanside, San Francisco, CA	California	Solids	250,000
Regional Water Recycling Plant No.1 (RP-1)	Ontario, CA	California	Solids	890,000
Calera Creek Water Recycling Plant	Pacifica, CA	California	Liquids	40,000
Palo Alto Regional Water Quality Control Plant *	Palo Alto, CA	California	Solids	236,000
City of Paso Robles Wastewater Treatment Plant	Paso Robles, CA	California	Solids	31,037
Ellis Creek Water Recycling Facility	Petaluma, CA	California	Liquids	65,000
Silicon Valley Clean Water *	Redwood City, CA	California	Solids	199,000
Regional Treatment Plant	Regional, Laguna Niguel, CA	California	Liquids	129,000
Riverside Water Quality Control Plant	Riverside, CA	California	Liquids	350,000
Sacramento Regional Wastewater Treatment Plant *	Sacramento, CA	California	Solids	1,480,000
E.W. Blom Point Loma Wastewater Treatment Plant	San Diego, CA	California	Liquids	2,200,000
San Jose-Santa Clara Regional Wastewater Facility *	San Jose, CA	California	Solids	1,500,000
City of San Leandro Water Pollution Control Plant	San Leandro, CA	California	Liquids	50,000
City of San Mateo & Estero M.I.D. Water Quality Control Plant	San Mateo, CA	California	Solids	150,000
Central Marin Sanitation Agency	San Rafael, CA	California	Liquids	104,250
City of Santa Cruz WTF - County Influent	Santa Cruz County, CA	California	Solids	160,000
City of Santa Cruz WTF - City Influent	Santa Cruz, CA	California	Solids	160,000
City of Santa Rosa, Laguna Treatment Plant	Santa Rosa, CA	California	Solids	230,000
Sausalito-Marin City Sanitary District	Sausalito, CA	California	Liquids	18,000

South Bay International Wastewater Treatment Plant	South San Diego, CA	California	Solids	1,600,000
Southeast Waste Pollution Control Plant *	Southeast San Francisco, CA	California	Solids	750,000
City of Sunnyvale Water Pollution Control Plant *	Sunnyvale, CA	California	Solids	153,000
Turlock Regional Water Quality Control Facility	Turlock, CA	California	Liquids	86,000
[Union City Basin] - Raymond A. Boege Alvarado WWTP	Union City, CA	California	Liquids	68,150
Vallejo Flood and Wastewater District Wastewater Treatment Plant	Vallejo, CA	California	Liquids	121,000
West County Wastewater District	West Contra Costa County, CA	California	Liquids	100,000
Central Marin Sanitation Agency - West Railroad	West Railroad, San Rafael, CA	California	Liquids	25,000
Windsor Wastewater Treatment, Reclamation, and Disposal Facility	Windsor, CA	California	Liquids	28,000
Winters - East Street Pump Station	Winters, CA	California	Liquids	7,286
Woodland Water Pollution Control Facility	Woodland, CA	California	Liquids	59,000
Parker Water and Sanitation District North Water Reclamation Facility	North, Parker, CO	Colorado	Liquids	35,000
Parker Water and Sanitation District South Water Reclamation Facility	South, Parker, CO	Colorado	Liquids	25,000
Stamford Water Pollution Control Authority (WPCA)	Stamford, CT	Connecticut	Solids	140,000
Seaford Wastewater Treatment Facility	Seaford, DE	Delaware	Solids	13,172
Altamonte Springs Regional Water Reclamation Facility	Altamonte Springs, FL	Florida	Liquids	95,000
Eastern Water Reclamation Facility	Eastern, Orange County, FL	Florida	Solids	195,299
Loxahatchee River Environmental Control District	Jupiter, FL	Florida	Liquids	90,000

MDWASD Central District WWTP	Key Biscayne, FL	Florida	Liquids	829,725
MDWASD North District WWTF	North Miami, FL	Florida	Liquids	776,150
Northeast Water Reclamation Facility	Northeast, Saint Petersburg, FL	Florida	Liquids	89,847
Northwest Water Reclamation Facility	Northwest, Orange County, FL	Florida	Solids	66,690
Northwest Water Reclamation Facility	Northwest, Saint Petersburg, FL	Florida	Liquids	94,218
MDWASD South District WWTF	South Miami, FL	Florida	Liquids	920,528
South Water Reclamation Facility	South, Orange County, FL	Florida	Solids	183,009
Hamlin Water Reclamation Facility	Southwest, Orange County, FL	Florida	Solids	50,000
Southwest Water Reclamation Facility	Southwest, Saint Petersburg, FL	Florida	Liquids	47,790
TPSmith Water Reclamation Facility	Tallahassee, FL	Florida	Liquids	212,065
Big Creek Water Reclamation Facility	Big Creek, Roswell, GA	Georgia	Liquids	189,593
Camp Creek Water Reclamation Facility	College Park, GA	Georgia	Liquids	73,821
South Columbus Water Resources Facility	Columbus, GA	Georgia	Solids	278,000
Johns Creek Environmental Campus	Johns Creek, Roswell, GA	Georgia	Liquids	84,486
Little River Water Reclamation Facility	Little River, Roswell, GA	Georgia	Liquids	12,818
RM Clayton Water Reclamation Center	RM Clayton, Atlanta, GA	Georgia	Liquids	294,660
South River Water Reclamation Center	South River, Atlanta, GA	Georgia	Liquids	105,160
Utoy Creek Water Reclamation Center	Utoy Creek, Atlanta, GA	Georgia	Liquids	70,887
Hilo Wastewater Treatment Plant	Hilo, HI	Hawaii	Liquids	16,257
Honouliuli Wastewater Treatment Plant	Honouliuli, Honolulu, HI	Hawaii	Liquids	300,000
Kailua Regional Wastewater Treatment Plant	Kailua, Honolulu, HI	Hawaii	Liquids	90,000

Sand Island Wastewater Treatment Plant	Sand Island, Honolulu, HI	Hawaii	Liquids	390,000
Wahiawa Wastewater Treatment Plant	Wahiawa, Honolulu, HI	Hawaii	Liquids	18,000
Waianae Wastewater Treatment Plant	Waianae, Honolulu, HI	Hawaii	Liquids	44,000
City of Coeur d'Alene Water Resource Recovery Facility	Coeur d'Alene, ID	Idaho	Solids	50,540
Lander Street Water Renewal Facility	Lander Street, Boise, ID	Idaho	Liquids	108,556
West Boise Water Renewal Facility	West Boise, ID	Idaho	Liquids	186,901
Glenbard Wastewater Authority	Glen Ellyn, IL	Illinois	Solids	86,000
Wheaton Sanitary District	Wheaton, IL	Illinois	Solids	63,000
Dillman Road WWTP	Bloomington, IN	Indiana	Liquids	56,090
City of Carmel WWTP	Carmel, IN	Indiana	Solids	86,000
Jeffersonville Downtown WWTP	Downtown, Jeffersonville, IN	Indiana	Liquids	25,000
North Water Reclamation Facility	North, Jeffersonville, IN	Indiana	Liquids	25,000
City of South Bend Wastewater Treatment Plant	South Bend, IN	Indiana	Liquids	130,000
City of Clinton	Clinton, IA	Iowa	Solids	29,300
Coralville Wastewater Treatment Facility	Coralville, IA	Iowa	Liquids	23,000
City of Marshalltown Water Pollution Control Plant	Marshalltown, IA	Iowa	Liquids	27,400
Muscatine STP	Muscatine, IA	Iowa	Solids	24,400
Ottumwa WPCF	Ottumwa, IA	Iowa	Liquids	25,529
Municipal Wastewater Treatment Plant No. 1 (Kaw Point)	Kaw Point, Kansas City, KS	Kansas	Solids	90,000
Lawrence Kansas River Wastewater Treatment Facility	Lawrence, KS	Kansas	Solids	80,000
Kansas City Treatment Plant #20	P20, Kansas City, KS	Kansas	Solids	35,000
Salina Wastewater Treatment Plant	Salina, KS	Kansas	Solids	47,000

Wolcott Wastewater Treatment Facility	Wolcott, Kansas City, KS	Kansas	Liquids	15,000
Morris Forman Water Quality Treatment Center	Louisville, KY	Kentucky	Solids	423,913
SWBNO East Bank Wastewater Treatment Plant	New Orleans	Louisiana	Liquids	333,400
SWBNO West Bank Wastewater Treatment Plant	New Orleans	Louisiana	Liquids	50,500
City of Bangor Wastewater Treatment Plant	Bangor, ME	Maine	Solids	40,000
Brunswick Sewer District	Brunswick, ME	Maine	Liquids	10,000
Lewiston Auburn Water Pollution Control Authority	Lewiston, ME	Maine	Liquids	60,000
Portland Water District (East End Wastewater Treatment Facility)	Portland, ME	Maine	Liquids	65,000
York Sewer District	York, ME	Maine	Liquids	10,000
Hagerstown Wastewater Treatment Plant	Hagerstown, MD	Maryland	Liquids	90,000
Marlay Taylor Water Reclamation Facility	Hollywood, MD	Maryland	Liquids	55,000
Deer Island Treatment Plant	Boston, MA	Massachusetts	Solids	2,400,000
Upper Blackstone Clean Water	Millbury, MA	Massachusetts	Liquids	250,000
City of Ann Arbor Wastewater Treatment Plant	Ann Arbor, MI	Michigan	Liquids	125,000
Jackson Wastewater Treatment Plant	Jackson, MI	Michigan	Solids	90,000
Grandville Clean Water Plant	Jenison, MI	Michigan	Solids	75,000
Mt. Pleasant WRRF	Mt. Pleasant, MI	Michigan	Liquids	21,690
Traverse City Regional Waste Water Treatment Plant	Traverse City, MI	Michigan	Liquids	30,623
City of Warren Wastewater Treatment Plant	Warren, MI	Michigan	Liquids	140,000

City of Mankato Water Resource Recovery Facility (WRRF)	Mankato, MN	Minnesot a	Solids	70,000
Red Wing Wastewater Treatment Facility	Red Wing, MN	Minnesot a	Solids	16,000
City Of Rochester MN Water Reclamation Plant	Rochester, MN	Minnesot a	Solids	120,000
St. Cloud Nutrient, Energy and Water Recovery Facility	St. Cloud, MN	Minnesot a	Liquids	120,000
7 C- Pascagoula Moss Point POTW (Vancleave, MS)	Jackson County, MS	Mississipp i	Liquids	34,333
2C-Gautier POTW (Vancleave, MS)	Jackson County, MS	Mississipp i	Liquids	19,008
Northeast Water Resource Recovery Facility	Northeast, Lincoln, NE	Nebraska	Liquids	60,000
Theresa Street Water Resource Recovery Facility	Theresa Street, Lincoln, NE	Nebraska	Liquids	240,000
Clark County Water Reclamation District (CCWRD) Flamingo Water Resource Center (FWRC)	Las Vegas, NV	Nevada	Liquids	990,000
City of Dover Wastewater Treatment Facility	Dover, NH	New Hampshir e	Liquids	30,000
Hall Street Wastewater Treatment Plant	Hall Street, Concord, NH	New Hampshir e	Liquids	45,000
Penacook Wastewater Treatment Facility	Penacook, Concord, NH	New Hampshir e	Liquids	4,000
South Monmouth Regional Sewerage Authority	Belmar, NJ	New Jersey	Solids	52,672
Cumberland County Utilities Authority	Bridgeton, NJ	New Jersey	Liquids	50,000
The Somerset Raritan Valley Sewerage Authority	Bridgewater, NJ	New Jersey	Liquids	130,000
Passaic Valley Sewerage Commission	Newark, NJ	New Jersey	Solids	1,500,000

Township of Ocean Sewerage Authority	Oakhurst, NJ	New Jersey	Liquids	50,000
Bayshore Regional Sewerage Authority	Union Beach, NJ	New Jersey	Solids	100,000
Ithaca Area Wastewater Treatment Facility	Ithaca, NY	New York	Liquids	90,000
City of Oswego Wastewater Treatment Plant	Oswego, NY	New York	Liquids	30,000
Johnnie Mosley Regional Water Reclamation Facility	Kinston, NC	North Carolina	Liquids	25,000
City of Wilson - Hominy Creek Water Reclamation Facility	Wilson, NC	North Carolina	Solids	50,000
Archie Elledge WWTP	Winston-Salem, NC	North Carolina	Liquids	92,000
Akron Water Reclamation Facility	Akron, OH	Ohio	Solids	365,000
City of Youngstown Wastewater Treatment Plant	Youngstown, OH	Ohio	Solids	174,000
DELCORA Western Regional Treatment Plant	Chester, PA	Pennsylvania	Liquids	220,000
Capital Region Water AWTF	Harrisburg, PA	Pennsylvania	Liquids	125,000
Penn State Water Treatment Facility	Penn State University Park	Pennsylvania	Liquids	16,000
City of Yankton Wastewater Treatment Facility	Yankton, SD	South Dakota	Liquids	20,000
Moccasin Bend WWTP	Chattanooga, TN	Tennessee	Liquids	400,000
M.C. Stiles Wastewater Treatment Facility	Memphis, TN	Tennessee	Liquids	300,000
DCWT Dallas	Dallas Central, Dallas, TX	Texas	Liquids	270,000
City of Gainesville Wastewater Treatment Plant	Gainesville, TX	Texas	Liquids	17,300
City of Garland Rowlett Creek WWTP	Garland, TX	Texas	Solids	200,000
Hollywood Road WWTP	Hollywood Road, Amarillo, TX	Texas	Liquids	60,000

River Road WWTP	River Road, Amarillo, TX	Texas	Liquids	140,000
South Laredo WWTP	South, Laredo, TX	Texas	Liquids	120,000
Southside Wastewater Treatment Plant (City of Dallas)	Southside, Dallas, TX	Texas	Liquids	421,700
Duck Creek Wastewater Treatment Plant	Sunnyvale, TX	Texas	Solids	186,000
DCWT White Rock	White Rock Central, Dallas, TX	Texas	Liquids	630,000
Wichita Falls Resource Recovery Facility	Wichita Falls, TX	Texas	Solids	90,000
SJRA WWTF No.1	Woodlands SJRA WWTF No. 1, TX	Texas	Liquids	65,000
SJRA WWTF No.2	Woodlands SJRA WWTF No. 2, TX	Texas	Liquids	70,000
SJRA WWTF No.3	Woodlands SJRA WWTF No. 3, TX	Texas	Liquids	15,000
Zacate Creek WWTP	Zacate Creek, Laredo, TX	Texas	Liquids	140,000
Central Valley Water Reclamation Facility	Central Salt Lake Valley, UT	Utah	Solids	600,000
Provo City Water Reclamation Facility	Provo, UT	Utah	Solids	115,000
City of Essex Junction Wastewater Treatment Facility	Essex Junction, VT	Vermont	Solids	30,000
Montpelier Water Resource Recovery Facility	Montpelier, VT	Vermont	Solids	10,100
South Burlington-Airport Parkway WWTF	South Burlington, VT	Vermont	Liquids	16,000
Aquia Wastewater Treatment Facility	Aquia, Stafford, VA	Virginia	Solids	100,000
Town of Hillsville Wastewater Treatment Plant	Hillsville, VA	Virginia	Solids	3,000
Little Falls Run Wastewater Treatment Facility	Little Falls Run, Stafford, VA	Virginia	Solids	50,000
City of Snohomish WWTP	Snohomish, WA	Washington	Liquids	10,150

City of Wheeling, Water Pollution Control Division	Wheeling, WV	West Virginia	Liquids	100,000
Wausau Waterworks Wastewater Treatment Facility	Wausau, WI	Wisconsin	Solids	44,000
Blue Plains Advanced Wastewater Treatment Tlant	Washington DC	District of Columbia	Liquids	2,000,000

Table S2. Other assays multiplexed with *C. auris* over the course of the study. HAV is hepatitis A virus, EVD68 is enterovirus D68, MPXV is mpox virus, HAdV_F is human adenovirus group F, HMPV is human metapneumovirus, Rota is rotavirus, HPIV is human parainfluenza virus. Dates are in month/day/year format. The fluorescent molecular is indicated after the name of the assay. FAM, 6-fluorescein amidite; HEX, hexachloro-fluorescein; Cy5, Cyanine-5; ROX, carboxyrhodamine. SCAN sites include the following wastewater treatment plants: South County Regional Wastewater Authority, City of Sunnyvale Water Pollution Control Plant, Palo Alto Regional Water Quality Control Plant, San Jose-Santa Clara Regional Wastewater Facility, Silicon Valley Clean Water, Oceanside Water Pollution Control Plant, Southeast Waste Pollution Control Plant, and Sacramento Regional Wastewater Treatment Plant, California. They are marked with an asterisk (*) In Table S1. Dates are in month/day/year format.

WWTPs A<br>(all other WWSCAN sites)	**9/11/23 - 10/1/23**<br><br>HAV (FAM)<br>C. auris (HEX)<br>EVD68 (FAM/HEX)<br>MPXV (Cy5)<br>HAdV_F (ROX)<br>HMPV (ATTO590)<br>Rota (ROX/ATTO590)	**10/2/23- 3/1/24**<br><br>HMPV (FAM)<br>C. auris (HEX)<br>EVD68 (FAM/HEX)<br>MPXV G2R_WA (Cy5)<br>HPIV (ROX/ATTO590)
WWTPs B<br>(SCAN sites)	**9/11/23 - 3/1/24**<br><br>HMPV (FAM)<br>C. auris (HEX)<br>EVD68 (FAM/HEX)<br>MPXV G2R_WA (Cy5)<br>HPIV (ROX/ATTO590)	

Table S3. Samples chosen to run two *C. auris* assays, for sequencing, and running with and without a RT (reverse transcription step). The location (city and state) and date where the samples were collected is provided.

**Samples run using two *C. auris* assays**	**Samples chosen for sequencing**	**Samples run using RT and no RT step**
Bangor, ME, 11/16/23<br>Bangor, ME, 9/28/23<br>Bangor, ME, 9/14/23<br>Bangor, ME, 1/8/24<br>Oakhurst, NJ, 9/26/23<br>Oakhurst, NJ, 1/4/24<br>Las Vegas, NV, 12/6/23<br>Las Vegas, NV, 12/8/23<br>Las Vegas, NV, 1/3/24	Bangor, ME, 11/16/23<br>Bangor, ME, 9/28/23<br>Bangor, ME, 9/14/23<br>Las Vegas, NV, 12/6/23	Bangor, ME, 11/16/23<br>Bangor, ME, 9/28/23<br>Bangor, ME, 9/14/23<br>Bangor, ME, 1/8/24<br>Oakhurst, NJ, 9/26/23<br>Oakhurst, NJ, 1/4/24<br>Las Vegas, NV, 12/6/23<br>Las Vegas, NV, 12/8/23<br>Las Vegas, NV, 1/3/24

Table S4. Forward and reverse primers, and probe sequences for detection of viral nucleic acids in this study. Primers and probes were purchased from Integrated DNA Technologies (IDT, Coralville, Iowa). All probes contained fluorescent molecules and quenchers (ZEN/3′ IBFQ). ZEN, a proprietary internal quencher from IDT; IBFQ, Iowa Black FQ.

Target	Primer/Probe	Sequence
BCoV	Forward	CTGGAAGTTGGTGGAGTT
	Reverse	ATTATCGGCCTAACATACATC
	Probe	CCTTCATATCTATACACATCAAGTTGTT
PMMoV	Forward	GAGTGGTTTGACCTTAACGTTTGA
	Reverse	TTGTCGGTTGCAATGCAAGT
	Probe	CCTACCGAAGCAAATG
Candida auris, Barber et al.[1]	Forward	CAG ACG TGA ATC ATC GAA TCT
	Reverse	TTT CGT GCA AGC TGT AAT TT
	Probe	AAT CTT CGC GGT GGC GTT GCA TTC A

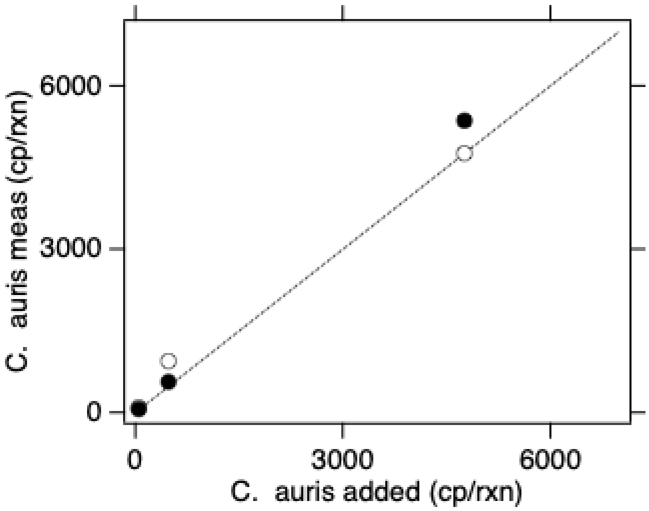

Figure S1. Multiplex assay performance. The concentration of the *C. auris* target in units of copies per reaction (cp/rxn) input and measured is provided. The white symbols are for reactions without the 7 background nucleic-acid targets and black symbols include high concentrations of the 7 other nucleic-acid targets. No amplification was found in any negative samples across 10 replicates. Error bars are standard deviations, if error bars cannot be seen, then they are smaller than the symbol. The line represents the 1:1 line.

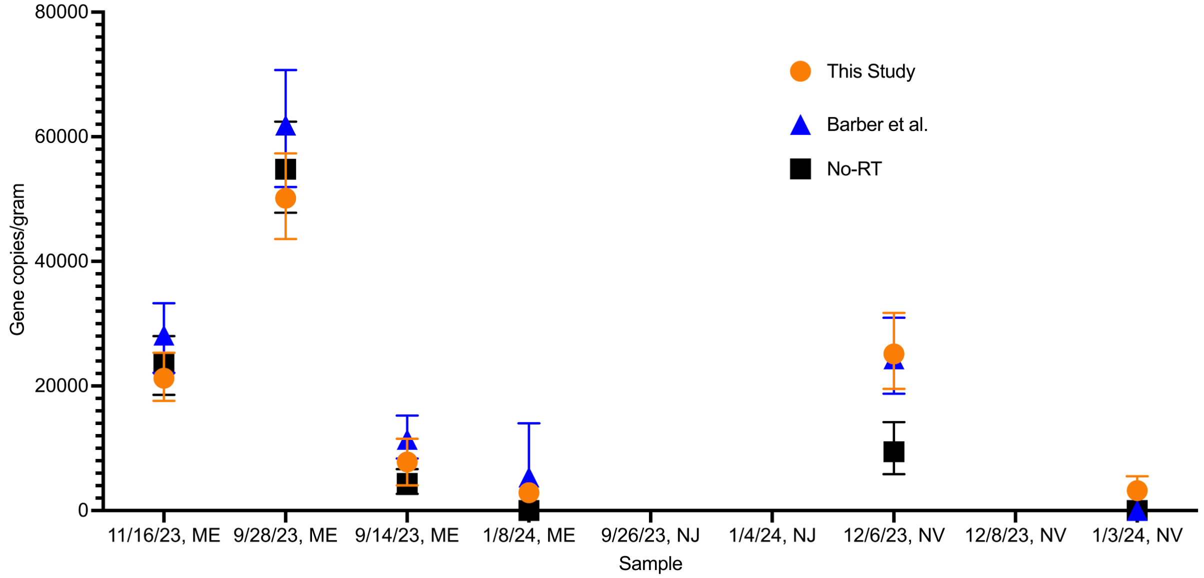

Figure S2. Comparison of Barber et al. assay to the assay used in this study. No significant differences were found between concentrations measured. Error bars represent standard deviations. Labels on x-axis refer to samples provided in Table S3.

Study Description	Environmental Sampling	Sample Treatment	Sample Reduction	Nucleic-acid Extraction	Reverse Transcription	PCR Amplification	Analysis
Study name: Candida au<br>Date: March 2024<br>Completed by: A. Boehm	Notes: Decribed in methods section	Notes: No sample treatment performed	Notes: Centrifugation was used, as described in the methods	Notes: Methods provided in paper.	Notes: Performed one-step RT-PCR	Notes: droplet digital PCR used	Notes: Provided in methods

Control Checklist	Environmental Sampling	Sample Treatment	Sample Reduction	Nucleic-acid Extraction	Reverse Transcription	PCR Amplification	
Step performed	☑	☐	☑	☑	☑	☑	
Step has control info	☐	☐	☐	☑	☑	☑	Negative controls
# of control replicates	0	na	0	2	2	2	
Control result reported	☐	☐	☐	☑	☑	☑	
Method for handling failed controls described	☐	☐	☑	☑	☑	☑	
Step has control info	☑	☐	☑	☐	☐	☐	Positive controls
Control identity described	☑	☐	☑	☑	☑	☑	
Control quantification method described	☑	☐	☑	☑	☑	☑	
# control replicates	internal control	na	6 to 10	6 to 10	6 to 10	6 to 10	
Control result reported	☑	☐	☑	☑	☑	☑	
Method for handling failed controls described	☑	☐	☑	☑	☑	☑	

Process checklist

**Environmental Sampling**	
Sample procedure	☑
Number of samples	☑
Sample amount, mean, range	☑
Sampling locations, dates, times	☑
Sample storage conditions	☑
**Sample Treatment**	
Treatment procedure	☐
Reagents	☐
**Sample Reduction**	
Reduction procedure	☑
Reagents	☑
Concentration factor	☑

**Nucleic-acid Extraction**	
Extraction procedure	☑
Volume or mass extracted, volume or mass obtained	☑
Extract storage conditions	☑
**Reverse Transcription**	
One- or two-step	☑
cDNA storage conditions (if 2 step)	☐
Reaction temperatures and times	☑
Reaction reagents and concentrations	☑
Priming method	☑
Reaction volume, added template amount	☑
RT efficiency assessment procedure (if 2-step)	☐
RT control description (if two-step)	☐
RT efficiency reported (if 2-step)	☐

**qPCR or dPCR**	
Target gene name, amplicon length	☑
Thermocycling temp and times	☑
Master mix composition: vendors, concentrations	☑
Additives: vendors, composition	☑
Template amount added, pre-treatment (if any)	☑
Primers: sequences, concentrations, vendors, references	☑
Amplicon confirmation method (probe, melt curve details, etc)	☑
Probe sequence, concentration, vendor, reference	☑
Instrumentation	☑
Inhibition assessment procedure	☑
Inhibition control description (if used)	☐
Number of samples tested and found inhibited	☐
Equivalent volume of sample analyzed	☑

**Analysis- dPCR**	
Threshold settings	☑
Technical replicates, number, well merging	☑
Partitions measured, number, mean, variance	☑
Partition volume	☑
Target copies per partition, mean, variance	☑
Program used for dPCR analysis	☑
Explanation of control results, example plots	☑
**Analysis- qPCR**	
Technical replicates, number, calculations	☐
Calibration standards, description, source	☐
Method of quantifying standards	☐
Calibration curve slope	☐
Calibration curve R2	☐
Lowest standard measured or 95% LOD	☐
Cq value determination methods	☐

Note to users: This checklist is provided as guidance for best practices for reporting, but is not meant to be prescriptive. Not all items in the check list will apply to all studies. Please see Borchardt et al. The Environmental

Version 3.0

This version maintained by Borchardt, Boehm, Salit, Noble, Wiggington, Spencer

Date: 18 October 2023

Figure S3. EMMI guidelines[2] checklist.

A

```
                 1                                                                                                            112
Positive ...     TCATCGAATCTTTGAACGCACATTGCGCCTTGGGGTATTCCCCAAGGCATGCCTGTTTGAGCGTGATGTCTTCTCACCAATCTTCGCGGTGGCGTTGCATTCACAAAATTAC
Sample 1, F      ----CGAaTCTTTGAACGCACATTGCGCCTTGGGGTATTCCCCAAGGCATGCCTGTTTGAGCGTGATGTCTTCTCACCAATCTTCGCGGTGGCGTTGCATTCACAAAATTAC
Sample 2, F      -----------------------GGCGCCTTGGGGTATTCCCCAAGGCATGCCTGTTTGAGCGTGATGTCTTCTCACCAATCTGCGCGGTGGCGTTGCATTCACAAAAGCAC
Sample 3, F      -----------------GCACATTGCGCCTTGGGGTATTCCCCAAGGCATGCCTGTTTGAGCGTGATGTCTTCTCACCAATCTTCGCGGTGGCGTTGCATTCACAAAATTAC
Sample 4, F      --------TCTTTGAACGCACATTGCGCCTTGGGGTATTCCCCAAGGCATGCCTGTTTGAGCGTGATGTCTTCTCACCAATCTTCGCGGTGGCGTTGCATTCACAAAATTAC

.....................................................................................................................................

                 113                                                                                        204
Positive ...     AGCTTGCACGAAAAAAATCTACGCTTTTTTTTCGTTTTGTTGTCGCCTCAAATCAGGTAGGACTACCCGCTGAACTTA--------------
Sample 1, F      AGCTTGCACGAAAAAAATCTACGCTTTTTTTTCGTTTTGTTGTCGCCTCAAATCAGGTAGGACTACCCGCTGAACTTA------------AG
Sample 2, F      AGCTTGCACGAAAAAAATCTACGCTTTTTTTTCGTTTTGTTGTCGCCTCAAATCAGGTAGGACTACCCGCTGAACTTANCATCATCAATAAG
Sample 3, F      AGCTTGCACGAAAAAAATCTACGCTTTTTTTTCGTTTTGTTGTCCCCTCAAATCAGGTAATACTACCCGCTGAACTTAACNNTATCAATAAG
Sample 4, F      AGCTTGCACGAAAAAAATCTACGCTTTTTTTTCGTTTTGTTGTCGCCTCAAATCAGGTAGGACTACCCGCTGAACTTA-----------AGN

.....................................................................................................................................
```

B

```
                 1                                                                                                            112
Positive ...     ---------------CAGCGAAATGCGATACGTAGTATGACTTGCAGACGTGAATCATCGAATCTTTGAACGCACATTGCGCCTTGGGGTATTCCCCAAGGCATGCCTGTTT
Sample 1, R      CATCGATGAATCACGCAGCGACATGCGATACGTAGTATGACTTGCAGACGTGAATCATCGAATCTTTGAACGCACATTGCGCCTTGGGGTATTCCCCAAGGCATGCCTGTTT
Sample 2, R      GATGAATG---TACGCAGCGAAATGCGATACGTAGTATGACTTGCAGACGTGAATCATCGAATCTTTGAACGCACATTGCGCCTTGGGGTATTCCCCAAGGCATGCCTGTTT
Sample 3, R      CATCGATGAANCACGCAGCGAACTTCGATACGTAGTATGACGTGCAGACGTGAATCCNCGAATCTTCGGACGCACATTGCCCCTTGGGGTATTCCCCAAGGCATGCCTGTTT
Sample 4, R      --------------------------------TAGTATGACTTGCAGACGTGAATCATCGAATCTTTGAACGCACATTGCGCCTTGGGGTATTCCCCAAGGCATGCCTGTTT

.....................................................................................................................................

                 113                                                                                       203
Positive ...     GAGCGTGATGTCTTTTCGCCTATGTTCANCGGTGGCGTTGCACTCACAAAACTNCA-----------------------------------
Sample 1, R      GAGCGTGATGTCTTCTCACCAATCTTCG-CGGTGGCGTTGCA-------------------------------------------------
Sample 2, R      GAGCGTGATGTCTATCAATG-----------------------------------------------------------------------
Sample 3, R      GAGCGTAATGTCTTCTCACCAATATTCG-CGGTGGCGCTGCA-------------------------------------------------
Sample 4, R      GAGCGTGATGTCTTCTCACCAATCTTCG-CGGTGGCGTTGCATTCACAAAATTACAGCTTGCACGAAAAAAATCTACGCTTTTTTTTCGTT

.....................................................................................................................................
```

Figure S4. Sequencing of positive samples compared to sequencing of known positive controls. Mismatches are highlighted in red, while unknown bases are yellow. Figure S2A shows the alignment of using our forward primers and the resulting sequence. The top line represents our positive control, and Samples 1-4 represent positive samples. Figure S2B shows the same results using our reverse primers and positive samples. Sample 1 is from Bangor, ME on 11/16/23, Sample 2 is from Bangor, ME on 9/28/23, Sample 3 is from Bangor, ME on 9/14/23, and Sample 4 is from Las Vegas, NV on 12/6/23.

a. California

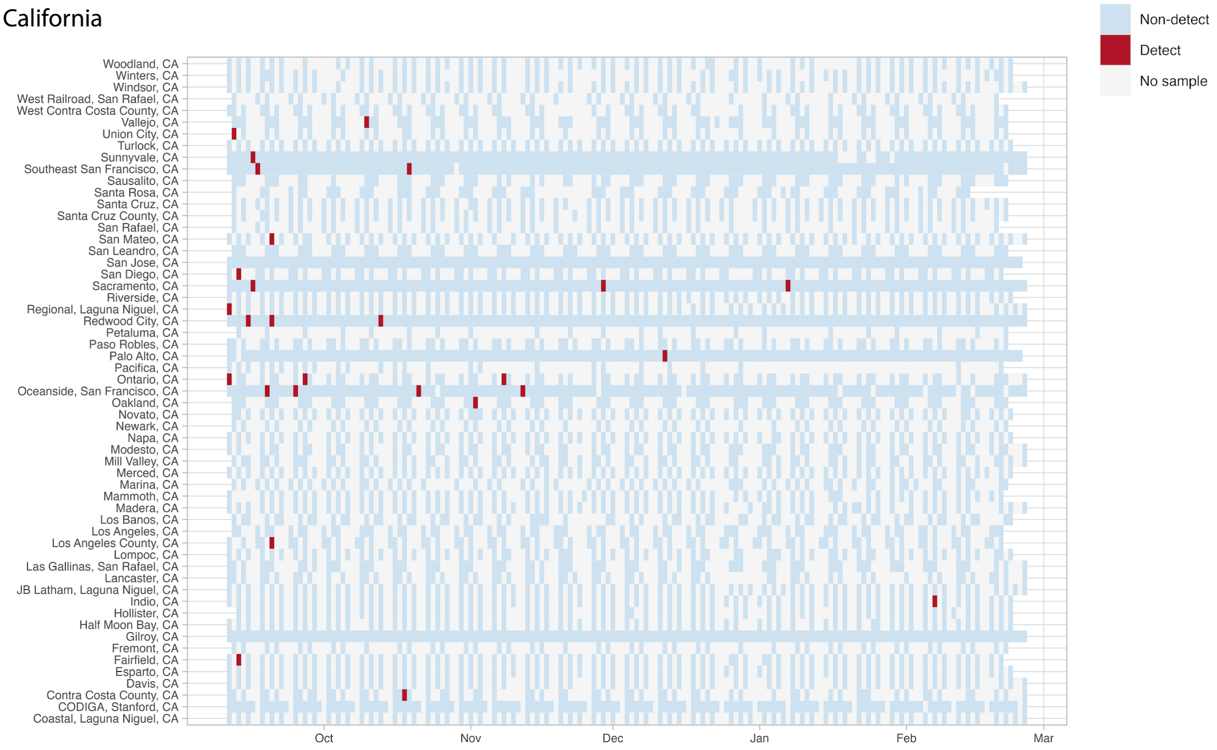

b. West

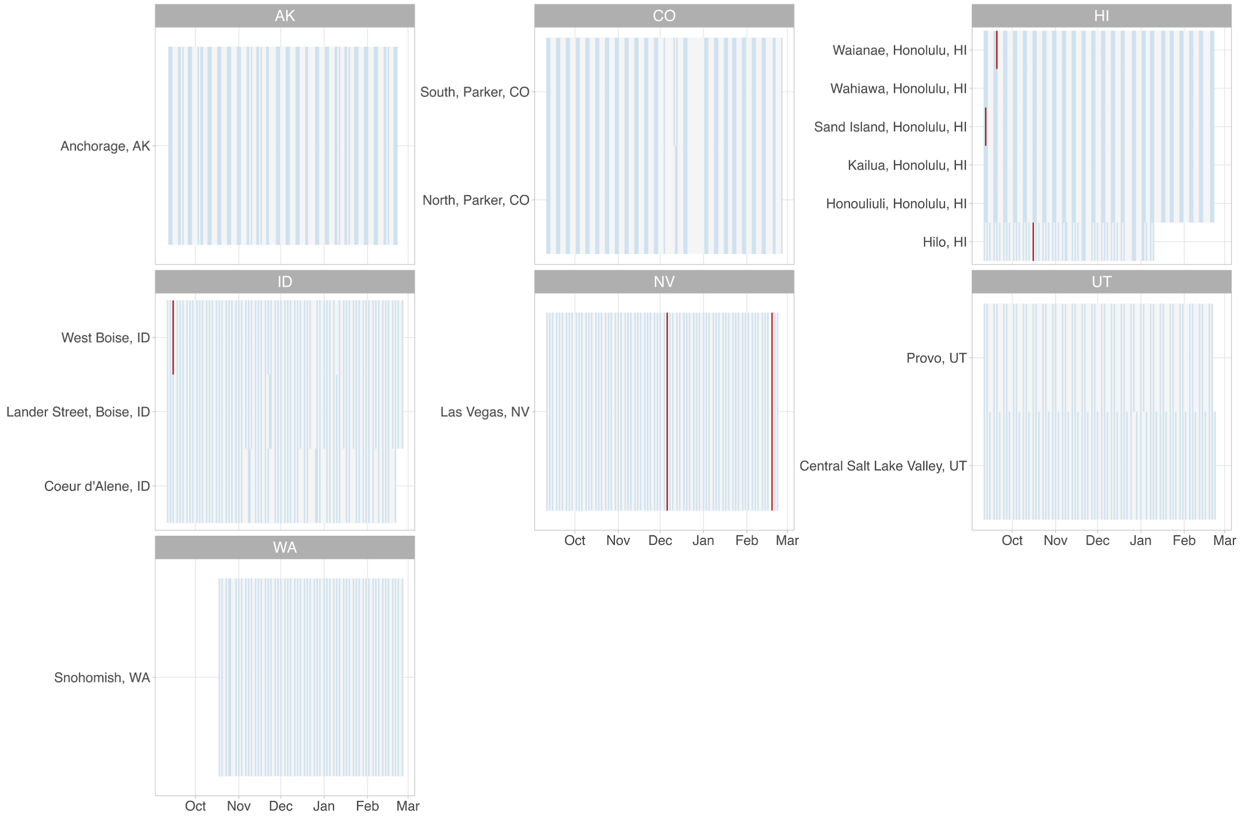

c. South

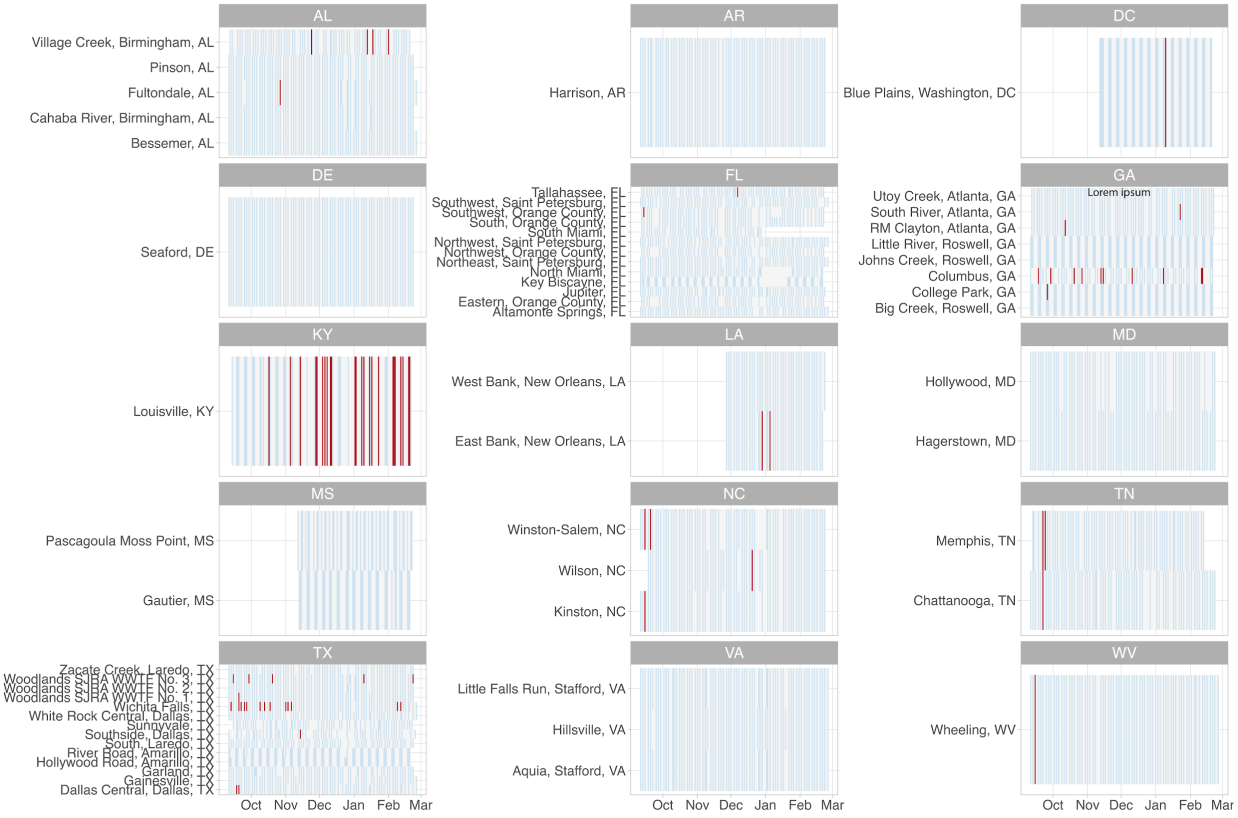
AL
Village Creek, Birmingham, AL
Pinson, AL
Fultondale, AL
Cahaba River, Birmingham, AL
Bessemer, AL
AR
Harrison, AR
DC
Blue Plains, Washington, DC
DE
Seaford, DE
FL
Tallahassee, FL
Southwest, Saint Petersburg, FL
Southwest, Orange County, FL
South, Orange County, FL
South Miami, FL
Northwest, Saint Petersburg, FL
Northwest, Orange County, FL
Northeast, Saint Petersburg, FL
North Miami, FL
Key Biscayne, FL
Jupiter, FL
Eastern, Orange County, FL
Altamonte Springs, FL
GA
Lorem ipsum
Utoy Creek, Atlanta, GA
South River, Atlanta, GA
RM Clayton, Atlanta, GA
Little River, Roswell, GA
Johns Creek, Roswell, GA
Columbus, GA
College Park, GA
Big Creek, Roswell, GA
KY
Louisville, KY
LA
West Bank, New Orleans, LA
East Bank, New Orleans, LA
MD
Hollywood, MD
Hagerstown, MD
MS
Pascagoula Moss Point, MS
Gautier, MS
NC
Winston-Salem, NC
Wilson, NC
Kinston, NC
TN
Memphis, TN
Chattanooga, TN
TX
Zacate Creek, Laredo, TX
Woodlands SJRA WWTF No. 3, TX
Woodlands SJRA WWTF No. 2, TX
Woodlands SJRA WWTF No. 1, TX
Wichita Falls, TX
White Rock Central, Dallas, TX
Sunnyvale, TX
Southside, Dallas, TX
South, Laredo, TX
River Road, Amarillo, TX
Hollywood Road, Amarillo, TX
Garland, TX
Gainesville, TX
Dallas Central, Dallas, TX
VA
Little Falls Run, Stafford, VA
Hillsville, VA
Aquia, Stafford, VA
WV
Wheeling, WV
Oct
Nov
Dec
Jan
Feb
Mar

d. Midwest

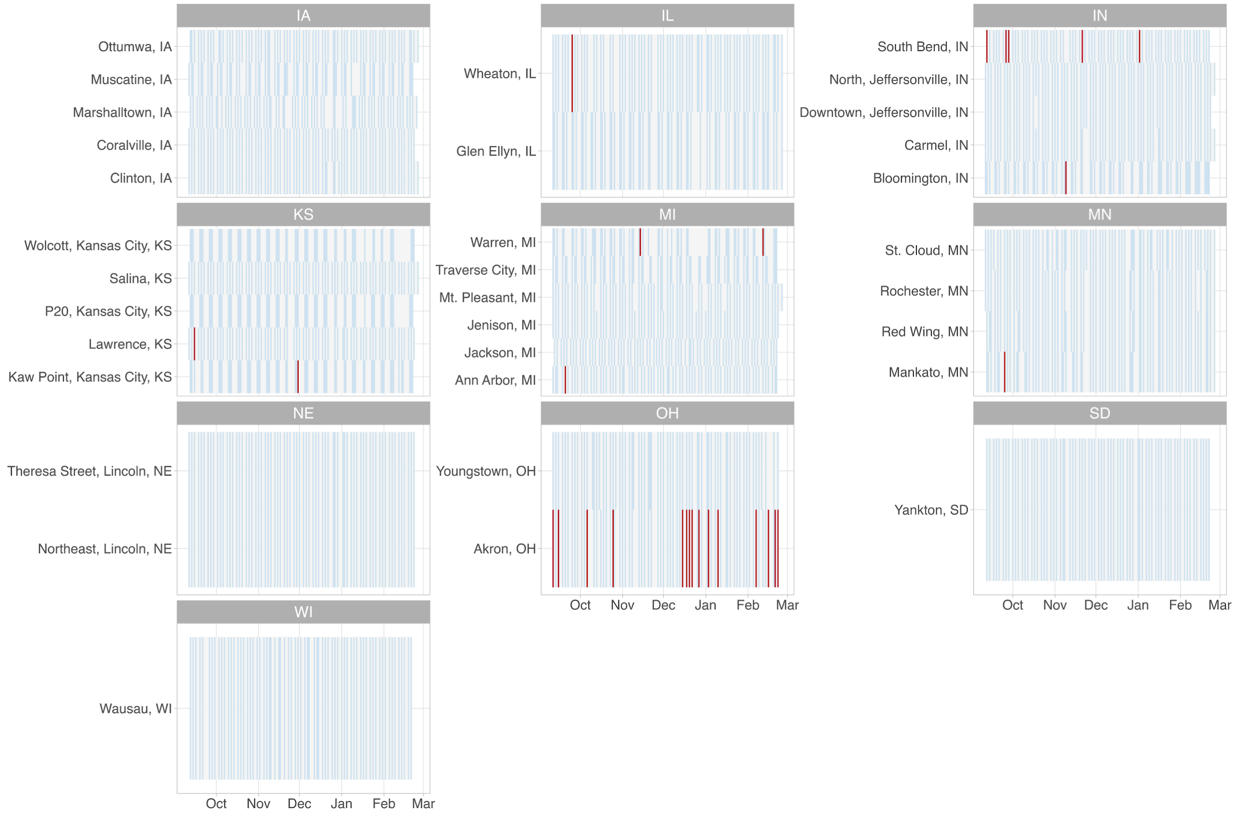

IA
Ottumwa, IA
Muscatine, IA
Marshalltown, IA
Coralville, IA
Clinton, IA
IL
Wheaton, IL
Glen Ellyn, IL
IN
South Bend, IN
North, Jeffersonville, IN
Downtown, Jeffersonville, IN
Carmel, IN
Bloomington, IN
KS
Wolcott, Kansas City, KS
Salina, KS
P20, Kansas City, KS
Lawrence, KS
Kaw Point, Kansas City, KS
MI
Warren, MI
Traverse City, MI
Mt. Pleasant, MI
Jenison, MI
Jackson, MI
Ann Arbor, MI
MN
St. Cloud, MN
Rochester, MN
Red Wing, MN
Mankato, MN
NE
Theresa Street, Lincoln, NE
Northeast, Lincoln, NE
OH
Youngstown, OH
Akron, OH
SD
Yankton, SD
WI
Wausau, WI
Oct
Nov
Dec
Jan
Feb
Mar

e. Northeast

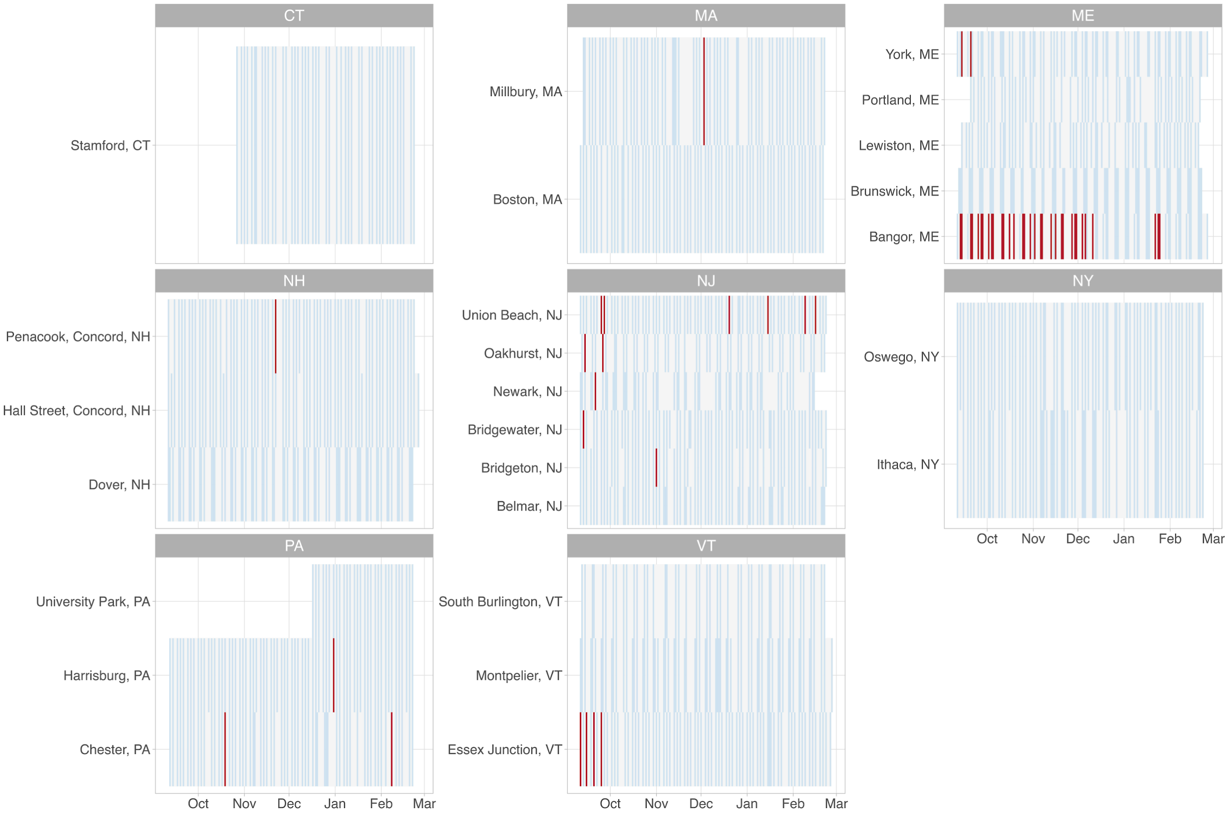

Figure S5. Heatmap of all national locations from September 11th, 2023 to March 1st, 2024. Graphs are organized by region and state. Figure S3a represents the locations in California, S5b is all states in the west, S5c is all states in the northeast, S5d is all states in the midwest and S5e is all states in the south.

References

(1) Barber, C.; Crank, K.; Papp, K.; Innes, G. K.; Schmitz, B. W.; Chavez, J.; Rossi, A.; Gerrity, D. Community-Scale Wastewater Surveillance of Candida Auris during an Ongoing Outbreak in Southern Nevada. *Environ. Sci. Technol.* **2023**, *57* (4), 1755–1763. https://doi.org/10.1021/acs.est.2c07763.

(2) Borchardt, M. A.; Boehm, A. B.; Salit, M.; Spencer, S. K.; Wigginton, K. R.; Noble, R. T. The Environmental Microbiology Minimum Information (EMMI) Guidelines: qPCR and dPCR Quality and Reporting for Environmental Microbiology. *Environ. Sci. Technol.* **2021**, *55* (15), 10210–10223. https://doi.org/10.1021/acs.est.1c01767.
